# Review of interoperability standards as data quality supporting elements

**DOI:** 10.1093/eurpub/ckac129.500

**Published:** 2022-10-25

**Authors:** P Doupi, F Estupiñán-Romero, M Comendeiro-Maaloe, R Launa, M Mäkinen, E Bernal-Delgado

**Affiliations:** 1 Data and Analytics Unit, Finnish Institute for Health and Welfare, Helsinki, Finland; 2 Data Sciences for Health Services and Policy, Institute for Health Sciences in Aragon, Zaragoza, Spain

## Abstract

WP6 has approached the target of supporting trustworthy secondary use of health and health care data through two operational objectives: developing the EHDS data quality assurance framework for real-world health data and developing the EHDS secondary use Semantic Interoperability Framework. For the latter objective, several interoperability standards were identified in accordance with the EHDS2 data life cycle and user's journey approach, hence focusing on data discoverability (at data source and variable levels), communication support across nodes and on development of common data models. Selection was based on active participation of WP6 leaders in various pertinent workshops and interactive activities, both in the framework of TEHDAS (Stakeholder and Project Forum meetings) as well as other relevant initiatives (e.g. the PHIRI project). Input was also sought from Commission representatives and EU-level regulatory authorities. In a first step, standards were catalogued based of features such as typology of interest, utility and application domains. In the next phase we organised virtual semi-structured interviews with key representatives of over 20 standards (incl. HL7, SNOMED, CDISC, DCAT, OMOP etc.). The focus of the interviews targeted experiences in standards’ actual use, challenges in their implementation, issues of maintenance and sustainability, as well as undergoing collaborations and developments. Sessions were recorded and subsequently the transcripts of discussion extracted automatically. The process of analysing interview materials is presently ongoing, using an adapted version of the Common Assessment Method for Standards and Specifications (CAMSS) v.4.0.0. toolkit. Interim results will be discussed within the Joint Action meeting activities in June 2022, to produce a version for wider stakeholder dialogue later in the fall. Results and recommendations generated through this process will also be presented for discussion with the workshop audience.

